# Refractory periods in SUNCT

**DOI:** 10.1007/s10194-012-0464-3

**Published:** 2012-06-09

**Authors:** Vimal Kumar Paliwal, Prabhat Singh, Achal Kumar, Sushil Kumar Rahi, Rakesh Kumar Gupta

**Affiliations:** 1 Department of Neurology, Sanjay Gandhi Postgraduate Institute of Medical Sciences, Lucknow, 226014 Uttar Pradesh India; 2 Department of Radiology, Sanjay Gandhi Postgraduate Institute of Medical Sciences, Lucknow, 226014 Uttar Pradesh India

Sir,

The refractory period in patients with SUNCT is studied by Pareja JA and group, and we deeply appreciate their contribution in today’s understanding of SUNCT. SUNCT has a resemblance with first division trigeminal neuralgia, but SUNCT can be distinguished by the presence of marked autonomic symptoms like conjunctival injection and tearing. However, we do not have clear understanding of the underlying pathophysiological divide between trigeminal neuralgia and SUNCT. Trigeminal autonomic reflex with central disinhibition of variable extent is believed to bring autonomic symptoms in an otherwise trigeminal neuralgia-like illness. So, the trigeminal neuralgia, SUNA and SUNCT may actually be a continuum [1]. It may still be worthwhile to differentiate trigeminal neuralgia from SUNCT, as the presence or absence of autonomic symptoms may actually point towards the underlying pathophysiology. However, trigeminal neuralgia (ophthalmic division) with florid autonomic features irrespective of refractory period as in our cases represents a spectrum of SUNCT [2]. Over a period of time, these patients may show variable attack severity and fluctuating duration of refractory period. Therefore, we referred to Cohn et al. [3] in our paper, not mistakenly though, as they recommended the application of absent refractory period to diagnostic criteria of SUNCT. We believe that refractory period may be present/relative in milder disease and may become absent with severe disease (saw tooth pattern or very frequent spontaneous attacks) [4, 5]. Once, refractory period is studied in a large cohort of SUNCT patients in respect to attack severity, disease chronicity, primary versus secondary disease or natural course versus on-medication course, it would be more authentic to apply absent refractory period to the diagnostic criteria of SUNCT.

## References

[1] Matharu MS, Goadsby PJ, Olesen J, Goadsby PJ, Ramdan NM, Tfelt-Hansen P, Welch KMA (2006). Sudden unilateral neuralgiform pain with conjunctival injection and tearing. The headaches.

[2] Paliwal VK, Singh P, Kumar A, Rahi SK, Gupta RK (2012). Short-lasting unilateral neuralgiform headache with conjunctival injection and tearing (SUNCT) with preserved refractory period: report of three cases. J Headache Pain.

[3] Cohen AS, Matharu MS, Goadsby PJ (2006). Short-lasting unilateral neuralgiform headache attacks with conjunctival injection and tearing (SUNCT) or cranial autonomic features (SUNA)—a prospective clinical study of SUNCT and SUNA. Brain.

[4] Pareja JA, Pareja J, Palomo T, Caballero V, Pamo M (1994). SUNCT syndrome. Repetitive and overlapping attacks, headache.

[5] Pareja JA, Sjaastad O (1994). SUNCT syndrome in the female. Headache.

